# Pattern of linear growth and progression of bone maturation for girls with early-onset puberty: A mixed longitudinal study

**DOI:** 10.3389/fped.2023.1056035

**Published:** 2023-03-09

**Authors:** Shuangyi Liu, Zhe Su, Lili Pan, Jinfeng Chen, Xiu Zhao, Li Wang, Longjiang Zhang, Qiru Su, Huiping Su

**Affiliations:** ^1^ Department of Endocrinology, Shenzhen Children’s Hospital, Shenzhen, China; ^2^ Department of Pediatric Research Institute, Shenzhen Children's Hospital, Shenzhen, China

**Keywords:** early-onset puberty, adolescence, bone age, linear growth, artificial intelligence

## Abstract

**Background and objective:**

With a worldwide trend to earlier age of onset of puberty, the prevalence of early-onset puberty (EP) among girls has increased. The impact of EP on the pattern of linear growth and bone maturation is unclear. Accordingly, the objective of our study was to describe this pattern for girls with EP in Shenzhen, China.

**Methods:**

A total of 498 untreated girls diagnosed with EP at Shenzhen Children's Hospital, China, between January 2016 and December 2021. A total of 1,307 anthropometric measurements and 1,307 left-hand radiographs were available for analysis. Artificial intelligence (AI) was used to determine bone age (BA). Participants were classified into groups according to chronological age (CA) and BA. The pattern of linear growth (height) and progression of bone maturation was described between groups using the Lambda-Mu-Sigma (LMS) method. Published height-for-CA and height-for-BA norm references for a healthy Chinese population were used for age-appropriate comparisons.

**Results:**

The mean CA of appearance of first pubertal signs (breast buds) was 8.1 ± 0.5 years. Compared to norm-referenced data, girls with EP were significantly taller at a CA of 7–10 years. This was followed by a slowing in linear growth after a CA of 10 years, with 71 girls with EP having already achieved their target adult height. From 7 to 10 years of BA, the linear growth was slower in the EP group compared to norm-reference values. This was followed by a period of catch-up growth at 11.2 years of BA, with growth curves approaching norm-referenced values. The BA progressed rapidly from 7 to 8 years of age in about half of the girls with EP (median ΔBA/ΔCA >1.9), slowing, thereafter, until the period of catch-up growth at 11.2 years of BA.

**Conclusions:**

BA provides a more reliable reference than CA to assess growth parameters among girls with EP. Our limited data set does indicate that EP does not negatively impact final adult height. Therefore, the growth curves from our study are relevant, providing a reference for pediatricians in this clinical population and, thus, preventing over-treatment for EP.

## 1. Introduction

The worldwide secular trend of earlier onset of puberty among girls has been confirmed over the last century (1–3). From 1977 to 2013, the age onset of breast development has, on average, occurred 3 months earlier every decade (4). However, the overall final adult height (FAH) of children and adolescents has been increasing year-by-year in Europe (5, 6), Africa (7), and Asia (8–10). In China specifically, the FAH for girls has increased from 158.2 cm in 1975 to 160.8 cm in 2019 (11, 12).

Early-onset puberty (EP) is defined as puberty that begins earlier than two standard deviations (2SD) or the 3rd percentile of the median age accepted as “normal” (13, 14). According to a cross-sectional study (10) on sexual maturation, EP in girls in China is defined as breast development that begins between 7.1 years and 9.2 years of age. Multiple research studies (15–17) have shown that most girls with EP can reach their mid-parental height (MPH). For girls with EP who progress from one pubertal stage to the next within 6 months or exhibit a bone age (BA) progression that exceeds 1 year per each chronological year, puberty is considered to be both “early” and “fast” (EFP) (18, 19). EFP may lead to an accelerated progression of BA and early epiphyseal closure, reducing the FAH. Considering the increasing incidence of EP among girls, periodical assessment and monitoring of FAH and BA would be necessary for individualized management of EP. To date, however, no longitudinal study of the linear growth and progression of bone maturation for girls with EP has been conducted in China to inform practice. Accordingly, our aim in this study was to describe the pattern of linear growth and bone maturation for girls with EP in south China to provide reference values.

## 2. Materials and methods

### 2.1. Study participants

Eligible participants for this study were untreated girls diagnosed with EP at Shenzhen Children's Hospital, China, between January 2016 and December 2021. Potential participants were identified from the hospital's database. The inclusion criteria were as follows: onset age of breast development between 7.1 years and 9.2 years of age; no drug therapies for EP; regular follow-up at intervals of 3–6 months; and BA assessments performed at least twice within a 6- to12-month interval. Exclusion criteria were: other causes of breast enlargement, including hamartoma, intracranial tumors, exogenous estrogen intake, or focal proliferative breast diseases; isolated telarche observed during the follow-up period; premature pubarche; birth weight that was small-for-gestational-age (SGA) or large-for-gestational-age (LGA); premature (PM), twin, or triplet birth; presence of other endocrine conditions, such as growth hormone deficiency, abnormal thyroid function, and abnormal adrenal cortex function; bone dysplasia, congenital dysplasia, cognitive impairment, or chronic health condition; long-term use of hormone therapy and use of Chinese herbal soup; psychomotor delay; abnormal nutritional status, with a body mass index (BMI) exceeding >120% of the 95th age-appropriate percentile (obesity) or <5th percentile (wasting) (20); family history of precocious puberty; and parental height >2 standard deviation (SD) or <−2 SD of the mean for the general population in China.

### 2.2. Statement of ethics

The study protocol was approved by the research ethics committee of Shenzhen Children's Hospital, China (20211036). All parents or guardians of participants provided written informed consent for use of their child's data for research.

### 2.3. Data variables and their measurements

Standardized anthropometric measurements were obtained, in the morning for all participants, by pediatric endocrinologists at Shenzhen Children's Hospital. Weight (to the nearest 0.01 kg), measured with the child in light clothing, and height (to the nearest 0.1 cm) were measured using calibrated and standardized apparatus following a standard procedure. The height of both parents was also obtained. The pubertal stage of breast development was assessed at every visit using Tanner's Stages of Puberty (21).

Plain radiographs of the left hand and wrist were obtained by radiologists; poor images were excluded. The radiographs were evaluated using an automated artificial intelligence (AI) system (Deepwise Artificial Intelligence Lab, Deepwise Inc., Beijing, China) and BA was assessed using the TW3-RUS method (22). Height-for-CA and height-for-BA were converted into standard deviation scores (SDS) according to the standardized growth curve for children and adolescents in China (23). Changes in height SDS (ΔHtSDS), body mass index (BMI), and the progression of bone maturation [increment of BA over increment of CA (ΔBA/ΔCA)] were calculated. The mid-parental height (MPH) was calculated for each participant, using Tanner's formula (21). The diagnostic criteria of FAH were defined as a growth velocity of <1 cm/year over the previous year or with a CA or BA ≥15 years.

### 2.4. Statistical analysis

Normality of the distribution of data was evaluated using the Shapiro-Wilk test. Normally distributed data were described as the mean ± SD, with the median (25th, 75th) reported for data with a non-normal distribution. Continuous variables were compared between groups using either an independent t-test or Mann–Whitney test, as appropriate for the data distribution, with a one-way analysis of variance (ANOVA) or Kruskal-Wallis test used, as appropriate, for comparison between multiple groups. Analyses were performed using SPSS Statistics (version 26.0, IBM Corp., Armonk, NY), with significance set at a *P*-value <0.05 was considered statistically significant. We removed data points when values were four standard deviations above or below the mean.

Curves of linear growth (height) and the progression of bone maturation were generated using the Lambda-Mu-Sigma (LMS) method (Chartmaker pro version). The LMS method (24) described the generalized data in each age group by three curves, representing the median (M), coefficient of variation (S), and skewness (L), the latter representing the Box-Cox power. The LMS is a well-accepted method for calculating standard growth curves. The goodness-of-fit of each model was tested and optimized using the Q-test for fit, detrended Q-Q plots, and comparisons between fitted and measured values. The 3rd and 97th percentile limits for each curve were calculated and plotted (Graph Pad Prism, version 8.0.2). The height velocity (HV)-for-CA curve and the HV-for-BA curve were generated referenced to the 50th percentile for height, fitted using the LMS method.

## 3. Results

### 3.1. Description of the study group

**Figure 1:**
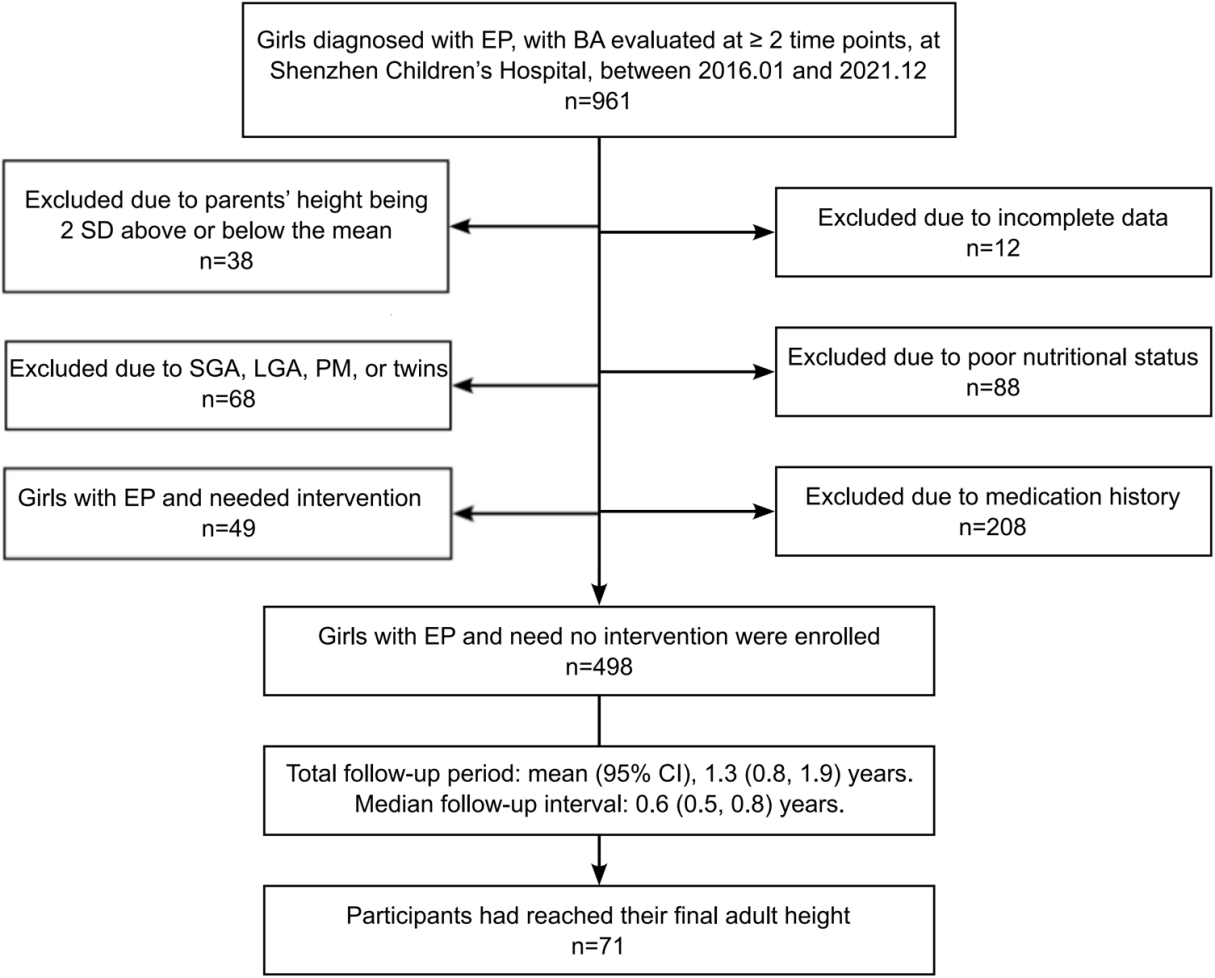
Flow chart for the selection of participants. EP, early-onset puberty; BA, bone age; SGA, small for gestational age; LGA, large for gestational age; PM, premature infants.

The flow chart for identifying girls with EP is shown in Figure 1. From the Shenzhen Children's Hospital database, 498 eligible participants and 49 treated girls with EP were identified (Table 1). Of the 498 eligible girls, 436 participants (88%) had a normal BMI and 62 (12%) were overweight. A total of 1,307 BA assessments and height measurements were available for analysis. The average onset age of breast development was 8.1 ± 0.5 (7.1, 9.2) years of age. The average recorded birth weight was 3.2 (3.0, 3.4) kg. The average paternal height was 170.0 (168.0, 174.0) cm and the average maternal height was 159.0 (155.0, 162.0) cm. Of the 498 participants, 71 had reached their FAH at the time of enrollment: median 162.0 (158.0, 165.1) cm, with a median difference to their MPH of 3.0 (1.5, 6.0) cm. The total follow-up time was 1.3 (0.8, 1.9) years, with a median follow-up interval of 0.6 (0.5, 0.8) years. Of the 498 participants, 41% had follow-up data at ≥3 time points.

**Table 1:** Comparison of the base data and anthropometric measurements between treated patients with EP and girls with EP who needed no intervention.

		Intervention (*n* = 49)	No intervention (*n* = 498)	*P*
Age of puberty onset, years		7.7(7.2, 8.1)	8.1 (7.7, 8.5)	<0.05
Time interval, years		0.6 (0.5, 1.0)	0.6 (0.5, 0.8)	>0.05
BMI		Overweight *n* = 8 (16.3%)	Overweight *n* = 62 (12.4%)	>0.05
MPH, cm		157.5 (155.0, 160.0)	158.5 (156.0, 160.7)	>0.05
Tanner 2 (*n*%)		52(53.1%)	694(53.1%)	>0.05
Tanner 3 (*n*%)		38 (38.8%)	484 (37%)	
Tanner 4 (*n*%)		8(8.2%)	101(7.7%)	
Tanner 5/menarche (*n*%)		—	28(2.1%)	
ΔBA/ΔCA	BA 7–10	2.4 (2.1, 2.9)	1.7 (1.2, 2.3)	<0.05
	BA ≥11	1.4 (0.9, 2.0)	0.8 (0.3, 1.4)	<0.05
PAH SDS	BA 7–10	0.2 (−0.2, 0.9)	0.5 (0.1, 1.1)	<0.05
	BA ≥11	−0.4 ± 0.9	0.3 ± 0.8	<0.05

*n*, number of measurements; FAH, final adult height; CA, chronological age; BA, bone age; MPH, mid-paternal height; PAH, predicted adult height; BMI, body mass index; SDS, stand deviation score. Normally distributed data were described as the mean ± SD, non-normal distribution data were described as median (25th, 75th).

### 3.2. Growth curves

**Figure 2:**
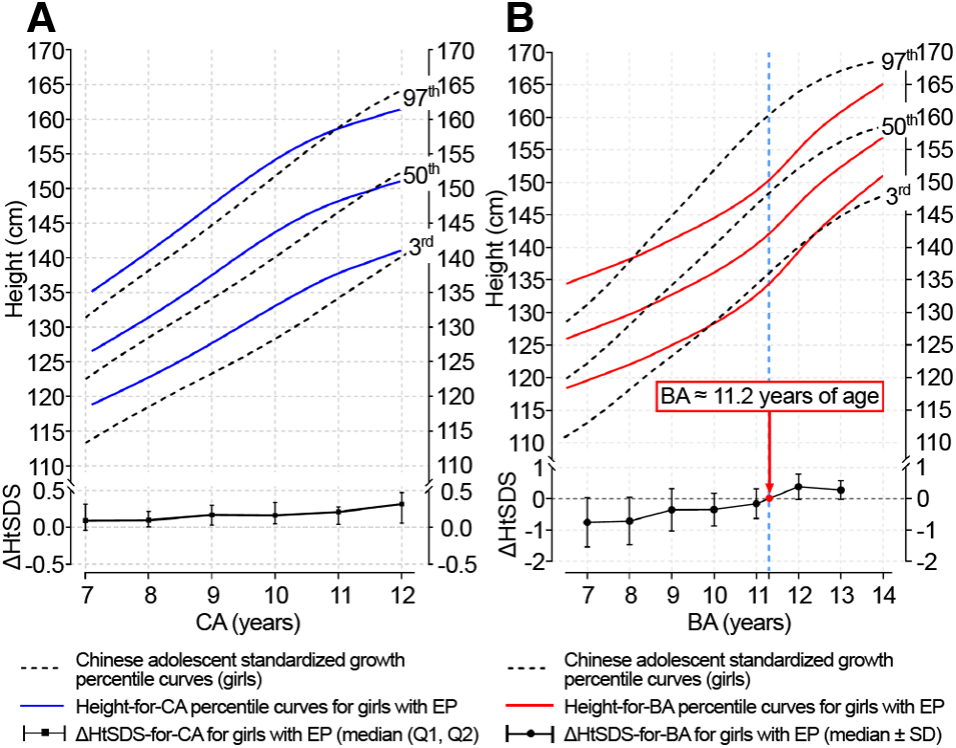
Height-for-CA/BA percentile curves and ΔHtSDS-for-CA/BA charts for girls with EP. CA, chronological age; BA, bone age; ΔHtSDS, changes in the height standard deviation score; EP, early-onset puberty.

The height-for-CA curves from 7 to 12 years of CA and height-for-BA curves from 7 to 14 years of BA are shown in Figure 2. There was no difference between the fitted and measured values for each CA group and BA group (*P *> 0.05). The fit of the curves is presented in Table 2. The fitted height-for-CA curves were compared, graphically, to standardized growth for Chinese adolescents (Figure 2A). From CA 7 to 10 years, the average height was significantly greater for the EP than the reference group (Table 3), with the growth progression for the EP group slowing after a CA of 10 years, with values similar to the standardized reference curves.

**Table 2:** Comparison between the P50th height (cm) of fitted values and measured values.

Table 2A							
Years	CA groups						
	*N* Measures	L	S	Measured value	Fitted value	Difference	*P*-value
7	59	−0.69	0.03	126.85	126.33	−0.52	0.99
8	329	−0.29	0.04	132.15	131.68	−0.47	-
9	509	0.54	0.04	136.8	137.25	0.45	-
10	295	1.27	0.04	143.7	143.85	0.15	-
11	95	0.95	0.04	148.15	148.16	0.01	-
12	20	0.53	0.04	150.2	151.12	0.92	-
Table 2B							
Years	BA groups						
	N Measures	L	S	Measured value	Fitted value	Difference	*P*-value
7	63	−0.69	0.03	127.55	127.20	−0.35	0.96
8	131	−0.58	0.03	129.30	129.72	0.42	—
9	256	−0.42	0.03	132.80	132.76	−0.04	—
10	305	−0.17	0.03	136.30	136.15	−0.15	—
11	255	−0.09	0.03	140.40	140.46	0.06	—
12	257	−1.43	0.03	147.00	146.70	−0.30	—
13	31	−4.01	0.03	151.50	152.36	0.86	—
14	9	−6.51	0.02	155.20	156.94	1.74	—

N Measures, number of measurements; CA, chronological age; BA, bone age; S, coefficient of variation; L, skewness.

**Table 3:** Comparison between the P50th height (cm) of fitted values and norm references.

CA group	Fitted value	Norm reference	Difference	*P*-value
7	126.33	122.5	3.83	0.002
8	131.68	128.5	3.18	
9	137.25	134.1	3.15	
10	143.85	140.1	3.75	
11	148.16	146.6	1.56	

CA, chronological age; Difference = Fitted value-Norm reference.

For the height-for-BA curves, linear growth slowed down from BA 7–11 years for the EP group compared to the standardized reference (Figure 2B). As BA progressed, the height growth curves for the EP group deviated from the standardized growth curves, shifting downward, with the height gap increasing gradually. A turning point, indicative of catch-up growth, did occur at a BA of 11.2 years, with the height-for-BA curves for the EP group approaching the standardized reference growth curves.

### 3.3. ΔHtSDS-for-CA and ΔHtSDS-for-BA

The ΔHtSDS-for-CA and ΔHtSDS-for-BA were grouped according to the mean CA and BA over two adjacent follow-up time points (Table 4), with 885 measurements available for each outcome. The ΔHtSDS-for-CA from CA 7–12 years and ΔHtSDS-for-BA from BA 7–14 years are plotted in Figure 2. When grouped by CA, no change in the trend of ΔHtSDS-for-CA values was noted across the CA groups (Figure 2A). However, when grouped by BA, the median ΔHtSDS-for-BA value was <0 (−0.75∼−0.15) from BA 7–11 years, indicative of an attenuation in growth of height-for-BA. As BA increased, an upward trend in the slow progression of ΔHtSDS-for-BA was observed, with the mean ΔHtSDS-for-BA value reaching a value of “0” at a BA of approximately 11.2 years, with values >0 thereafter, indicative of a catch-up growth of height (Figure 2B).

**Table 4:** ΔHtSDS-for-CA and ΔHtSDS-for-BA of EP girls.

Years	ΔHtSDS-for-CA		ΔHtSDS-for-BA	
	N Measures	ΔHtSDS	N Measures	ΔHtSDS
7 (6.50∼)	20	0.10 (−0.04,0.32)	20	−0.75 ± 0.79
8 (7.50∼)	215	0.10 (0.01,0.22)	86	−0.71 ± 0.76
9 (8.50∼)	380	0.17 (0.03,0.30)	170	−0.35 ± 0.68ab
10 (9.50∼)	205	0.17 (0.05,0.34)b	216	−0.35 ± 0.53ab
11 (10.50∼)	50	0.21 (0.04,0.28)	237	−0.15 ± 0.47abcd
12 (11.50∼)	15	0.32 (0.06,0.48)	143	0.39 ± 0.41abcde
13 (12.50∼)	—	—	14	0.28 ± 0.30bcd
*P*-value	—	<0.05	—	<0.05

Grouped by median CA or BA of the samples. As an example, for the 7-year-old group, the mean CA or BA was ≥6.5 years but <7.5 years, while for the 8-year-old group the mean CA or BA was ≥7.5 years but <8.5 years.

N Measures, number of measurements; CA, chronological age; BA, bone age; ΔHtSDS, changes in height standard deviation score.

^a^ compared to the 7-year-old group, *P* < 0.05.

^b^ compared to the 8-year-old group, *P *< 0.05.

^c^ compared to the 9-year-old group, *P* < 0.05.

^d^ compared to the 10-year-old group, *P* < 0.05.

^e^ compared to the 11-year-old group, *P* < 0.05.

### 3.4. Bone maturation curves

**Figure 3:**
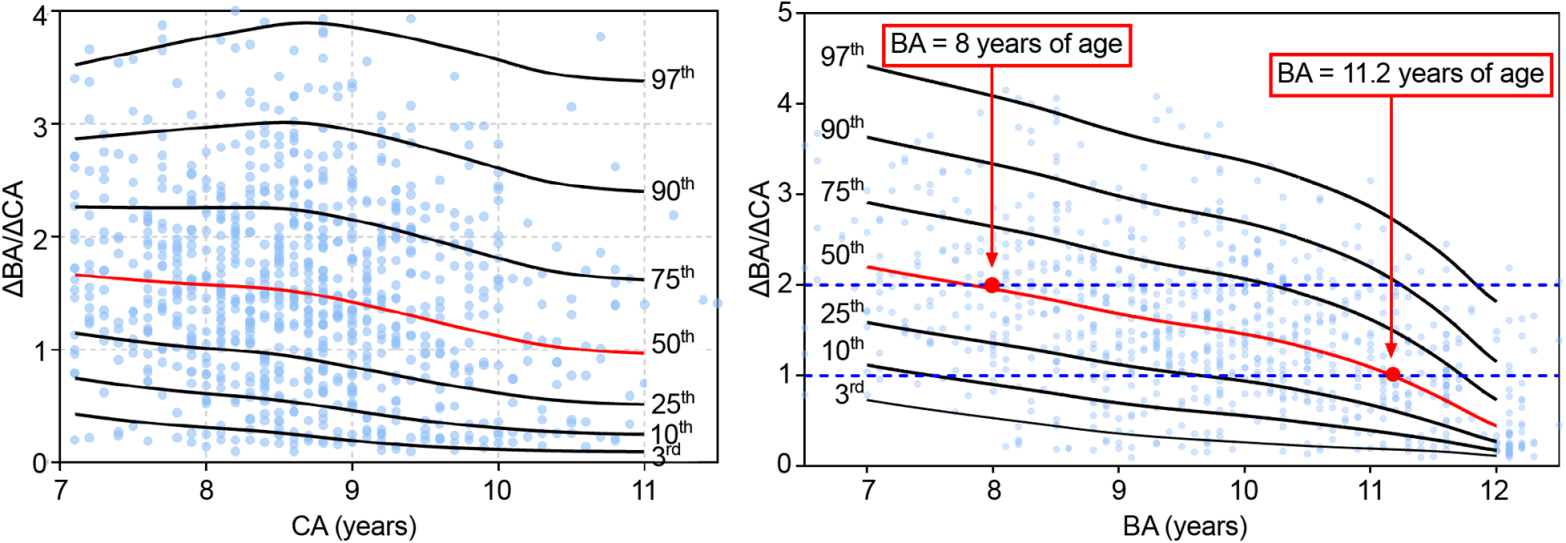
Δba/ΔCA-for-CA/BA percentile curves for girls with EP. CA, chronological age; BA, bone age; EP, early-onset puberty.

The ΔBA/ΔCA value indicates the rate of increment of BA per unit of CA over a 6 month to 1 year interval. Values were grouped by CA and BA. The following percentile values were calculated from the smoothed curves: P3rd, P10th, P25th, P50th, P75th, P90th, and P97th. Percentile ΔBA/ΔCA-for-CA curves for the 7–11 years CA interval and ΔBA/ΔCA-for-BA curves for the 7–12 years BA interval, constructed using the LMS method, are shown in Figure 3, with no significant difference between the fitted and measured values (*P *> 0.05; Table 5). The values of ΔBA/ΔCA-for-CA were scattered among all CA groups, with no observable trend compared to BA groups (Figure 3). As for ΔBA/ΔCA-for-BA, values of ΔBA/ΔCA decreased gradually while BA increased over the 7–12 year interval. Nearly half of the participants had significantly progressive BA advancement (median ΔBA/ΔCA, 1.96 in the 8-year-old group) between the BA interval of 7–8 years. As the rate of bone maturation slowed down, the progression in BA also decreased, compared to CA, after a BA of 11.2 years (median ΔBA/ΔCA, 0 at a BA of 11.2 years; Figure 3).

**Table 5:** Comparison between P50th ΔBA/ΔCA of fitted values and measured values .

Table 5A							
Years	CA groups						
	N Measures	L	S	Measured value	Fitted value	Difference	*P*-value
7	59	0.58	0.5	1.7	1.66	−0.04	0.92
8	296	0.52	0.59	1.53	1.57	0.04	—
9	368	0.48	0.68	1.51	1.42	−0.09	—
10	126	0.41	0.79	1.08	1.12	0.04	—
11	36	0.36	0.84	1.02	0.97	−0.05	—
Table 5B							
Years	BA groups						
	N Measures	L	S	Measured value	Fitted value	Difference	*P*-value
7	61	0.52	0.45	2.08	2.2	0.12	0.94
8	125	0.57	0.49	1.97	1.96	−0.01	—
9	209	0.6	0.53	1.64	1.68	0.05	—
10	226	0.58	0.57	1.55	1.46	−0.09	—
11	151	0.45	0.64	1.21	1.09	−0.12	—
12	113	−0.03	0.73	0.56	0.45	−0.11	—

N Measures, number of measurements; CA, chronological age; BA, bone age; S, coefficient of variation; L, skewness.

## 4. Discussion

To our knowledge, this is the first study to describe the pattern of linear growth (height) and progression of bone maturation for girls with EP in China. Our data, thus, can provide a reference to inform clinical assessment and individualized management of this clinical population, preventing over-treatment. The growth curves presented for height and bone maturation are based on a retrospective longitudinal clinical study at single center in China, including the data for 498 participants and 1,307 left hand and wrist radiographs to determine BA. Objectivity and accuracy of BA assessment was ensured by using an AI system and the LMS method, as previously described (25–28).

Our findings underline the importance of BA in identifying a rapid progression in linear growth and puberty development. For EP, BA, therefore, provides a better predictor than CA to evaluate growth and bone maturity. In our study sample, in the early stage of puberty, BA progressed faster than linear growth, with an attenuation in linear growth identified when referenced to BA. Therefore, the predicted FAH might be underestimated, which could lead to over-treatment. Generally, the identification of EFP is based on clinical manifestation, including a shorten interval between pubertal stages and progressive bone maturity. Noteworthy, the accelerated progression in bone maturation, one of the most significant traits of EFP, measured as a ΔBA/ΔCA >1 (18), might not be generalizable to the entire EFP population across different BA ranges. Based on our results, the threshold of BA progression for EFP diagnosis should be set according to the different BA groups.

Multiple studies (29–32) have shown that most girls with EP present growth patterns comparable to those reported in our study, including a younger age for onset of puberty and linear growth, reaching the MPH as their FAH. The height-for-CA curves in our study indicated that girls with EP had a higher average height than the norm reference between the ages of 7–10 years, with a subsequent slowing in linear growth to reach the same FAH as the standardized reference group. A retrospective observational study on the growth of 170 Israeli children and 335 Polish children (33) indicated that, generally, the growth of children and adolescents is a process of reaching the genetic target height (MPH). The greater the difference between the percentile childhood height and the MPH, the earlier the initiation of puberty will be. Comparable to our height-for-CA curves, the FAH in that study approached the normal range of MPH over time.

The earlier age of onset of puberty was not associated with a reduced FAH in our study group. In recent decades, with a global secular trend to earlier onset of puberty (4, 34, 35), there has also been a chronological trend of accelerated and advanced bone maturation, worldwide (16, 17, 36–37). However, the overall FAH has been increasing year-by-year. In China, the mean FAH has increased by 6.1 cm from 1985 to 2019 (15). An American longitudinal study of 380 girls, followed between birth and a mean age of 15.5 years, indicated a comparable FAH between girls with EP and those with late onset puberty (29). A meta-analysis conducted by Cheng et al. (38) also demonstrated that girls with EP have a greater potential for growth than girls with late-onset puberty (39–41). As shown in our study, girls with EP who had reached their FAH were, on average, 3.0 cm taller than their predicted MPH. Our growth curves indicate that girls with EP are likely to reach their target FAH. This information might help pediatric endocrinologists in more precisely evaluating the growth of girls with EP and prevent over-treatment of this population.

For diagnosis of EFP, clear consensus or standards regarding evaluation of bone maturation have yet to be clearly defined based on sufficient evidence. BA gives a more accurate assessment of growth potential and pace of puberty than CA. In our study, no trend was identified for the ΔBA/ΔCA-for-CA, with no association to the height-for-CA curves. Therefore, this variable does not provide useful information about bone maturation or growth potential. By comparison, the ΔBA/ΔCA-for-BA variable did provide a description of the pattern of linear growth and progression of bone maturation for girls with EP. In our study, a significantly earlier progression in bone maturation (median ΔBA/ΔCA >1.9) was identified in almost half of the participants at a BA of 7–8 years. Thereafter, the progression in BA slowed gradually, with the BA increment lagging CA. At a BA of approximately 11.2 years, the ΔBA/ΔCA leveled off (value of 1) as the ΔHtSDS for BA continues to improve. Therefore, the fact that BA progression leads CA in the early stage of puberty, especially at a BA of 7–8 years, does not always imply an impairment in linear height progression. This is why the predicted FAH is commonly underestimated in the early stage of puberty in girls with EP which, combined with a rather rapid BA progression, can lead to excessive testing and over-treatment. Based on our findings, dynamic observations of BA may be warranted in girls with EP and, thus, identification of the progression in bone maturation should be incorporated in the overall follow-up assessment of this clinical population. With increasing evidence, cut-off values for significant progressing in BA should be set for the different BA intervals.

According to existing guidelines (18), progressive EFP should be suspected when BA progresses at a rate >1 year per year (ΔBA/ΔCA >1). The majority of participants in our study had a ΔBA/ΔCA >1 before a BA of 11 years, with no indication for drug therapy. Of note, more than half of the participants in our study group had a ΔBA/ΔCA >2 at a BA of 7–8 years. This significant progression in bone maturation during this period may be considered as a normal variation and, therefore, it might be more reasonable to judge the progression of bone maturation as a function of BA rather than CA. For example, a ΔBA/ΔCA >1.9 could be considered as normal at a BA of 7 years, while a ΔBA/ΔCA >1 might be of clinical concern after a BA of 11 years. A prospective, multi-center, case-control study in China (42), including 260 girls with EFP, between 2012 and 2014, demonstrated that BA progression in girls with EFP girls could accelerate to a mean ΔBA/ΔCA of 1.9 at a mean BA of 9.2 years, which was between the 50th and 75th percentile for our results. Similarly, a retrospective study (42) of 135 girls in Turkey reported a mean ΔBA/ΔCA of up to 2.0 at a mean BA of 11.1 years for girls with EFP (>75th percentile of our result), while a mean ΔBA/ΔCA of 1.1 at a mean BA of 10.3 years for girls with EP (<50th percentile for our result). Consistent with our findings, girls with EP reached their target MPH. A ΔBA/ΔCA >50th percentile in our results may be indicative of possible EFP, while a ΔBA/ΔCA >75th percentile would be highly suggestive of a fast progression in bone maturation. Further studies with a larger number of participants will be needed to define specific ΔBA/ΔCA cutoff values.

The limitations of our study need to be acknowledged. Foremost is the retrospective design of the study, using cross-sectional data collected over a 5-year period. Therefore, causation between EP and linear growth and progression of BA cannot be defined. Moreover, the data were obtained from a population at a single center. Lastly, the sample size is relatively small and, combined with the single site, may not be representative of girls with EP in the entire Chinese population.

In summary, we provide growth curves for girls with EP in China, showing an initial attenuation in linear growth (height) referenced to BA, with a catch-up period after a BA of 11.2 years. A slowing in the progression of bone maturation was identified at a BA of 7–12 years. Based on our findings, we propose that the progression of bone maturation to predict growth in girls with EP should be referenced to BA and not CA. This might inform an individualized management of EP and prevent over-treatment. Further research, with a larger and more diverse study sample, is required to defined valid cut-off values to assess EP and monitor follow-up.

## Data Availability

The raw data supporting the conclusions of this article will be made available by the authors, without undue reservation.
